# Effect of correcting iron deficiency on the risk of serious infection in heart failure: Insights from the IRONMAN trial

**DOI:** 10.1002/ejhf.3504

**Published:** 2024-10-25

**Authors:** Paul W. Foley, Paul R. Kalra, John G.F. Cleland, Mark C. Petrie, Philip A. Kalra, Ian Squire, Philip Campbell, Callum Chapman, Patrick Donnelly, Fraser Graham, Andrew Hannah, Ninian N. Lang, Iain Matthews, Stephen J. Leslie, Pierpaolo Pellicori, Sue Piper, Robin Ray, Hernry O. Savage, Chales Spencer, John Walsh, Yuk‐Ki Wong, Ian Ford, on behalf of the IRONMAN Study Group, Paul Kalra, Elena Cowan, Charlotte Turner, Rosalynn Austin, Rebeca Lane, Paula Rogers, Paul Foley, Badri Chandrasekaran, Eva Fraile, Lynsey Kyeremeh, Fozia Ahmed, Mark Petrie, Lorraine McGregor, Joanna Osmanska, Fraser Graham, Ninian Lang, Barbara Meyer, Faheem Ahmad, Joanna Osmanska, Iain Squire, Jude Fisher, Philip Kalra, Christina Summersgill, Katarzyna Adeniji, Rajkumar Chinnadurai, Andrew Ludman, Lisa Massimo, Clare Hardman, Daisy Sykes, Peter Cowburn, Sarah Frank, Simon Smith, Alan Japp, Mohamed Anwar, Beth Whittington, Alison Seed, Robin Ray, Vennessa Sookhoo, Sinead Lyons, Abdallah Al‐Mohammad, Janet Middle, Kay Housley, Andrew Clark, Jeanne Bulemfu, Christopher Critoph, Victor Chong, Stephen Wood, Benjamin Szwejkowski, Chim Lang, Jackie Duff, Susan MacDonald, Rebekah Schiff, Patrick Donnelly, Thuraia Nageh, Swapna Kunhunny, Mark Petrie, Roy Gardner, Marion McAdam, Elizabeth McPherson, Prithwish Banerjee, Eleanor Sear, Nigel Edwards, Jason Glover, Pierpaolo Pellicori, Clare Murphy, Justin Cooke, Charles Spencer, Mark Francis, Iain Matthews, Hayley McKie, Andrew Marshall, Janet Large, Jenny Stratford, Piers Clifford, Sara Tavares, Christopher Boos, Philip Keeling, Debbie Hughes, Aaron Wong, Deborah Jones, Alex James, Rhys Williams, Stephen Leslie, Jim Finlayson, Piers Clifford, Andrew Hannah, Philip Campbell, John Walsh, Jane Quinn, Callum Chapman, Susan Piper, Sheetal Patale, Preeti Gupta, Victor Sim, Lucy Knibbs, Kristopher Lyons, Lana Dixon, Colin Petrie, Yuk‐ki Wong, Catherine Labinjoh, Simon Duckett, Ian Massey, Henry Savage, Sofia Matias, Jonaifah Ramirez, Charlotte Manisty, Ifza Hussain, Rajiv Sankaranarayanan, Gershan Davis, Samuel McClure, John Baxter, Eleanor Wicks, Jolanta Sobolewska, Jerry Murphy, Ahmed Elzayat, Alastair Cooke, Jay Wright, Simon Williams, Amal Muthumala, Parminder Chaggar, Sue Webber, Gethin Ellis, Mandie Welch, Sudantha Bulugahapitiya, Thomas Jackson, Tapesh Pakrashi, Ameet Bakhai, Vinodh Krishnamurthy, Reto Gamma, Susan Ellery, Charlotte Manisty, Geraint Jenkins, Gladdys Thomas, Angus Nightingale

**Affiliations:** ^1^ The Great Western Hospital Swindon UK; ^2^ Department of Cardiology Portsmouth Hospitals University NHS Trust Portsmouth UK; ^3^ College of Medical, Veterinary and Life Sciences, University of Glasgow Glasgow UK; ^4^ Faculty of Science and Health University of Portsmouth Portsmouth UK; ^5^ School of Cardiovascular and Metabolic Health, University of Glasgow Glasgow UK; ^6^ Department of Renal Medicine Salford Royal Hospital, Northern Care Alliance NHS Foundation Trust Salford UK; ^7^ NIHR Biomedical Research Centre, Glenfield Hospital Leicester UK; ^8^ Royal Gwent Hospital Newport UK; ^9^ Chelsea and Westminster Hospital London UK; ^10^ Ulster Hospital Southeastern Health and Social Care Trust Belfast UK; ^11^ Aberdeen Royal Infirmary Aberdeen UK; ^12^ Queen Elizabeth University Hospital Glasgow UK; ^13^ Wansbeck General Hospital Ashington UK; ^14^ Cardiac Unit Raigmore Hospital Inverness UK; ^15^ Kings College Hospital London UK; ^16^ St Georges Hospital London UK; ^17^ Mid and South Essex NHS Foundation Trust Basildon UK; ^18^ New Cross Hospital Wolverhampton UK; ^19^ Nottingham University Hospital, Nottingham City Hospital Nottingham UK; ^20^ St Richard's Hospital Chichester UK; ^21^ Robertson Centre for Biostatistics, University of Glasgow Glasgow UK

**Keywords:** Heart failure, Iron deficiency, Infection, Hospitalization, Mortality

## Abstract

**Aims:**

Concerns exist that intravenous (IV) iron might increase the risk of infections. The IRONMAN trial provided an opportunity to investigate whether giving IV ferric derisomaltose (FDI) to patients with heart failure and iron deficiency alters the rate of hospitalization or death due to infections.

**Methods and results:**

IRONMAN was a randomized trial of IV FDI versus usual care in patients with symptomatic heart failure, left ventricular ejection fraction (LVEF) ≤45%, and transferrin saturation (TSAT) <20% or ferritin <100 μg/L. Infection was a pre‐specified, blindly‐adjudicated, safety endpoint. The primary analysis of interest was infection as the main reason for hospitalization or death, using first and recurrent events analyses. The composite primary event of interest tended to be lower in those randomized to FDI when analysed as first (hazard ratio [HR] 0.79, 95% confidence interval [CI] 0.62–1.01, *p* = 0.055) or recurrent event (rate ratio 0.85, 95% CI 0.64–1.13, *p* = 0.089). The composite results were driven by fewer hospitalizations for infection (HR 0.76, 95% CI 0.49–0.98, *p* = 0.032), with 5% fewer patients (absolute reduction) experiencing such an event if assigned to FDI. Similar trends were observed for recurrent events (HR 0.82, 95% CI 0.62–1.10). Further analyses suggested that the reduction in hospitalizations due to infection with FDI was restricted to patients with TSAT <20%.

**Conclusions:**

In patients with heart failure and a reduced LVEF, correction of iron deficiency is not associated with an increased risk of hospitalization or death from infection, and may reduce such events, especially when TSAT is <20%.

Clinical Trial Registration: ClinicalTrials.gov, NCT02642562.

## Introduction

Iron deficiency is common in patients with heart failure and clinical trials suggest that correction with high‐dose intravenous (IV) iron improves symptoms and quality of life and reduces the risk of heart failure hospitalization.^1^, ^2^, ^3^ There is a theoretical risk that iron supplements might increase the risk or severity of infection. Iron is essential for bacterial growth and laboratory studies have shown that exogenous iron may enhance bacterial proliferation^4^ and inhibit host defence pathways, including impeding phagocytic function.^5^ Yet, iron deficiency itself may adversely impact the hosts T cell, B cell and antibody responses to infections.^6^ Observational studies of IV iron have produced conflicting results.^7^, ^8^ A meta‐analysis (across many clinical indications) suggested an increased risk of infections with IV iron (risk ratio 1.17; 95% confidence interval [CI] 1.04–1.31) but with marked variation in the definition and reporting of infections among trials.^9^ As most trials did not pre‐specify infection as an endpoint, infections were probably under‐reported. A need for prospective randomized trials of IV iron, including infection as a pre‐specified endpoint, has been highlighted.^9^, ^10^ The PIVOTAL trial demonstrated no increased risk of infection with higher IV iron dosing versus lower dosing regimens in patients on haemodialysis.^11^ However, further data with comparison to usual care without IV iron and in other conditions are needed. This is particularly relevant for heart failure, given the complex relationship between decompensated heart failure and infection.^12^

IRONMAN (effectiveness of intravenous iron treatment versus standard care in patients with heart failure and iron deficiency) was a randomized trial comparing IV ferric derisomaltose (FDI) versus usual care in patients with heart failure, reduced left ventricular ejection fraction (LVEF) and iron deficiency.^13^ The primary outcome was a composite of hospitalization for heart failure or cardiovascular death. However, hospitalization and death due to infection were important pre‐specified safety endpoints. Given that a large part of the trial was conducted during the COVID‐19 pandemic, we also evaluated COVID‐19 infections.

## Methods

### Trial design

Briefly, IRONMAN was an investigator‐initiated, prospective, randomized, open‐label, blinded endpoint (PROBE), event‐driven trial funded by the British Heart Foundation (grant award CS/15/1/31175). Pharmacosmos supplied FDI and further financial support. The trial design and the overall trial results have been published.^3^, ^13^

### Patients

Patients in the United Kingdom, aged ≥18 years, with new or established symptomatic heart failure, evidence of iron deficiency (serum ferritin <100 μg/L or transferrin saturation [TSAT] <20%) and LVEF ≤45% in the preceding 24 months were included. Patients were required either to have a current or recent (within 6 months) heart failure admission or, for patients not fulfilling either of these criteria, have raised plasma concentrations of natriuretic peptides (N‐terminal pro‐B‐type natriuretic peptide >250 ng/L in sinus rhythm or >1000 ng/L in atrial fibrillation or B‐type natriuretic peptide >75 pg/ml or 300 pg/ml, respectively). Patients were excluded if they had active infection, serum ferritin >400 μg/L or haemoglobin <9.0 g/dl or >13 g/dl for women or >14 g/dl for men. Randomization was stratified by trial site and recruitment context and patients provided written informed consent.

The dose of FDI was calculated according to an individual patient's weight and haemoglobin, up to a maximum of 2000 mg per infusion.^13^ For all patients, follow‐up was planned at 4 weeks, 4 months, and then every 4 months. To maintain iron repletion in patients randomized to IV FDI, a further infusion was given at follow‐up visits if ferritin was <100 μg/L or TSAT was <25% (provided ferritin ≤400 μg/L).

### Outcomes

The trial primary and secondary endpoints have been published.^3^ Hospitalization and death from infection were pre‐specified safety endpoints. As a large part of the IRONMAN trial was conducted during the COVID‐19 pandemic, hospitalizations and deaths due to COVID‐19 were collected and reported. A clinical endpoints committee, blinded to treatment allocation, adjudicated all deaths and unplanned hospitalizations for both the main and contributory causes of events.

For this paper, the primary analysis of interest was infection as the main reason for hospitalization or death, using first and recurrent events approaches. We also report an analysis of recurrent events for hospitalizations or deaths where infection was the main or contributory reason.

Infections were subcategorized to the most affected systems specified. These categories of infection were: non‐COVID respiratory, COVID‐19, skin and soft tissue, urinary and other (including those where primary sites of infection was uncertain).

Two further exploratory analyses were conducted. Firstly, censoring data at 1 year, to further reduce the effect of the COVID‐19 pandemic on the trial's conduct, which prevented patients assigned to IV FDI from being re‐dosed for long periods.^3^ Secondly, given the growing evidence that the effect of IV iron on clinical events is greater when TSAT is <20%,^14^ we performed subgroup analyses of patients with baseline TSAT <20% and TSAT ≥20% for time‐to‐first event outcomes.

### Statistical analysis

All analyses were carried out in the validly randomized population (one patient was randomized in error and was excluded from all analyses). Recurrent events were analysed by the method of Lin *et al*.,^15^ with mean frequency functions displayed using the method of Ghosh and Lin.^16^ The association between baseline characteristics and risk for infection was assessed in a recurrent events model for fatal and hospitalized infection. Time‐to‐first‐event fatal or hospitalized infections were analysed using Cox proportional hazards models and displayed graphically as a cumulative incidence function adjusting for deaths not included in the outcome analysed. All analyses included treatment group and recruitment context as covariates. A Clinical Endpoints Committee adjudicated infection events. All analyses used SAS version 9.4 (SAS Institute Inc., Cary, NC, USA) or R version 4.3.2 (R Foundation for Statistical Computing, Vienna, Austria).

### Role of the funding source

The funders of the study had no role in the trial design, data collection, data analysis, data interpretation, or writing of the report. The first draft of this paper was prepared by the first two and last authors. It was reviewed by all authors, who made the decision to submit the paper.

## Results

A total of 1137 patients were validly randomized to IV FDI or usual care and were followed up for a median of 2.7 (interquartile range 1.8–3.6) years (maximum of 5.4 years). Twelve patients assigned to IV FDI withdrew consent and a further five were lost to follow‐up compared, respectively, to seven and six for usual care.

Of those assigned to IV FDI, 217 received a single infusion, 226 two infusions, 81 three infusions, and 35 between four and nine infusions. Of those assigned to usual care, 95 (17%) received IV iron outside the protocol, with 48 patients receiving IV iron in the first year of follow‐up. Of those 95 patients, 69 received a single infusion, 21 received two infusions, and five received between three and five infusions.

Baseline characteristics which were associated with greater rates of hospitalization due to infection include higher LVEF, higher systolic blood pressure, impaired renal function, the presence of diabetes mellitus, higher baseline ferritin (but not TSAT), anaemia and being in New York Heart Association class III or IV rather than II (*Table* *1*).

**Table 1:** Baseline characteristics by infection status during the trial (with or without infection as the main cause of hospitalization or death)

Infection‐related event during follow‐up	≥ 1 Infection	No Infection	*p*‐value
*n*	266	871	
Recruitment context			
Inpatient	46 (17)	118 (14)	0.19
Recent admission^a^	51 (19)	157 (18)	
Outpatient	169 (64)	596 (68)	
Age (years)	74 (68–79)	73 (66–79)	0.36
Women	61 (23)	239 (27)	0.99
NYHA class >II	138 (52)	351 (40)	0.0045
Ischaemic aetiology	159 (60)	488 (56)	0.29
Previous admission for HF	173 (65)	488 (56)	0.069
De novo HF	19 (7)	96 (11)	0.28
History of AF	119 (45)	415 (48)	0.16
History of ACS	138 (52)	439 (50)	0.93
Hypertension	143 (54)	469 (54)	0.53
Diabetes	143 (54)	378 (43)	0.0072
BMI (kg/m^2^)	28 (25–32)	29 (25– 33)	0.13
Heart rate (bpm)	70 (62– 80)	69 (60, 79)	0.014
LVEF (%)	35 (28–40)	32 (25–37)	0.012
SBP (mmHg)	121 (108–135)	118 (106–132)	0.0058
TSAT (%)	16 (11–19)	15 (11–19)	0.46
TSAT <20%	195 (75)	646 (76)	0.19
Ferritin (μg/L)	52 (31–88)	49 (29–85)	0.031
eGFR (ml/min/1.73 m^2^)	47 (36–63)	53 (39–70)	<0.001
Haemoglobin (g/dl)	11.9 (11.1–12.8)	12.1 (11.2–12.9)	0.026

Data are summarized as median (interquartile range) for continuous variables and *n* (%) for categorical variables, with *p*‐values for univariate tests of significance of characteristic in predicting infection events in a Cox proportional hazards model.

ACS, acute coronary syndrome; AF, atrial fibrillation; BMI, body mass index; eGFR, estimated glomerular filtration rate (Levey calculation); HF, heart failure; LVEF, left ventricular ejection fraction; NYHA, New York Heart Association; SBP, systolic blood pressure; TSAT, transferrin saturation.

^a^ Within preceding 6 months.

**Figure 1:**
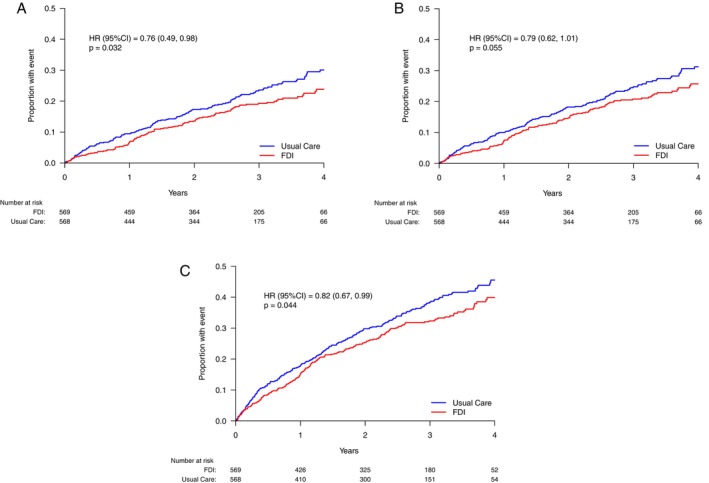
Cumulative incidence curves for selected infection outcomes. (*A*) Cumulative incidence functions for the outcome of first hospitalization due to infection as the main cause. (*B*) Cumulative incidence functions for the outcome of first hospitalization or death due to infection as the main cause. (*C*) Cumulative incidence functions for the outcome of first hospitalization due to infection as the main or contributory cause. CI, confidence interval; FDI, ferric derisomaltose; HR, hazard ratio.

*Table* *2* shows the infection‐related events for both arms of the trial. When evaluated using first event analysis, there were fewer hospitalizations or deaths with infection as the main cause in patients assigned to FDI (hazard ratio [HR] 0.79, 95% CI 0.62–1.01, *p* = 0.055) (*Figure* *1B*). Fewer patients were hospitalized with infection as the main cause if assigned to FDI (*n* = 111; 20%) compared to usual care (*n* = 140; 25%) (HR 0.76, 95% CI 0.49–0.98, *p* = 0.032) (*Figure* *1A*). Also, fewer patients were hospitalized with infection as the main or contributary cause in those assigned to FDI (*n* = 189; 33%) compared to usual care (*n* = 221; 39%) (HR 0.82, 95% CI 0.67–0.99, *p* = 0.032) (*Figure* *1C*).

**Table 2:** Hospitalizations with, and deaths due to, infection

Outcome	Usual care (*n* = 568)	FDI (*n* = 569)	HR or RR (95% CI)	*p*‐value
**First events**	***n* (% with an event)**	***n* (% with an event)**		
Hospitalization or death with infection as the main cause (first event)	146 (26)	120 (21)	0.79 (0.62–1.01)	0.055
Hospitalization with infection as the main cause (first event)	140 (25)	111 (20)	0.76 (0.59–0.98)	0.032
Hospitalization with infection as the main or contributory cause (first event)	221 (39)	189 (33)	0.82 (0.67–0.99)	0.044
Deaths with infection as the main cause	28 (5)	34 (6)	1.22 (0.74–2.02)	0.43
Deaths due to infection as the main cause				
COVID‐19	9	5		
Other respiratory	9	13		
Skin and soft tissue	1	2		
Urinary	1	3		
Other	8	11		
**Recurrent events**	***n* (rate per 100 patient‐years)**	***n* (rate per 100 patient‐years)**		
Hospitalizations with infection as the main cause (recurrent events)	213 (14)	175 (12)	0.82 (0.62–1.10)	0.19
Hospitalization with infection as the main cause				
COVID‐19	20	14		
Other respiratory	68	42		
Skin and soft tissue	34	19		
Urinary	36	33		
Other	55	67		
Hospitalization or death with infection as the main cause (recurrent events)	223 (15)	189 (13)	0.85 (0.64–1.13)	0.26
Hospitalizations with infection as the main or contributory cause (recurrent events)	420 (28)	345 (23)	0.82 (0.66–1.03)	0.089

Numbers and % with events for first events and numbers of events and rates/100 patient‐years for recurrent events with hazard ratios (HR) (for first events) and rate ratios (RR) (for recurrent events) with associated 95% confidence intervals (CI) and *p*‐values comparing ferric derisomaltose (FDI) with usual care.

For patients randomized to FDI, 120 (21%) were hospitalized with infection as the main cause or died due to infection: 84 patients had a single event, 20 patients two events, and 16 patients three or more events. For patients randomized to usual care, 146 (26%) had an infection related event: 105 patients had a single event, 24 patients two events, and 17 patients three or more events. With FDI, eight infections occurred within 28 days of inclusion compared to four patients assigned to usual care. There were similar numbers of fatal infections in each randomized group (*Table* *2*).

**Figure 2:**
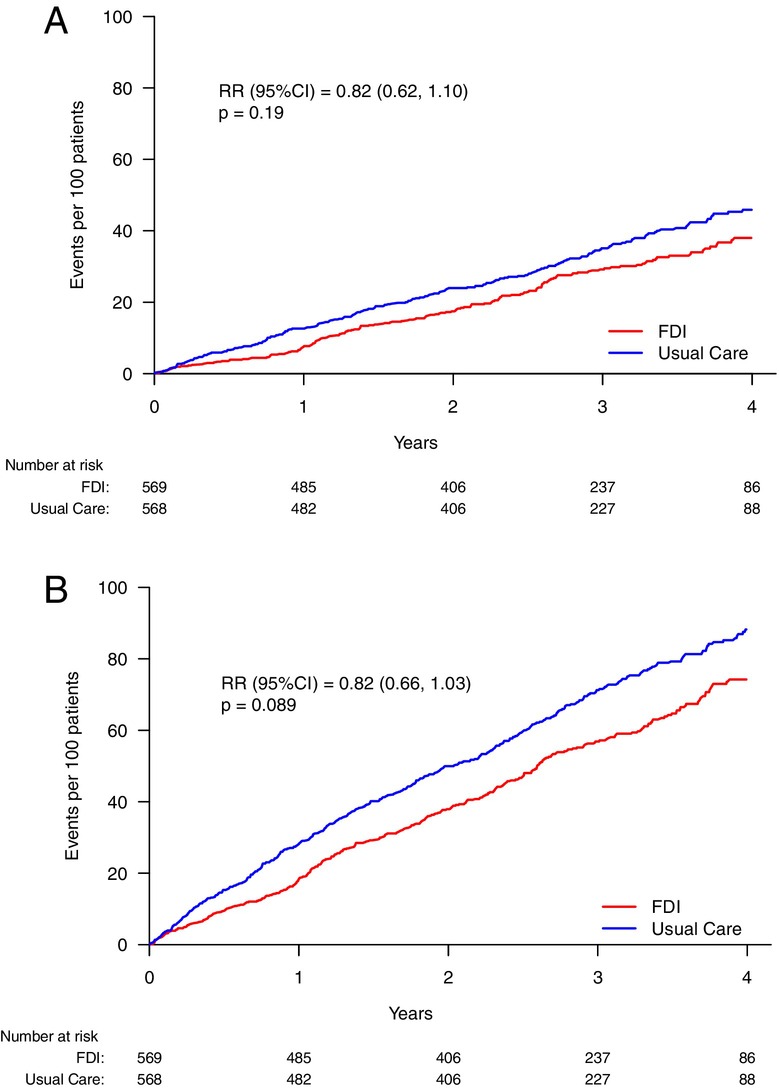
Estimated mean frequency functions for selected infection outcomes. (*A*) Recurrent hospitalization with infection as the main cause. (*B*) Recurrent hospitalization due to infection as the main or contributory cause. CI, confidence interval; FDI, ferric derisomaltose; RR, risk ratio.

There were numerically fewer recurrent events with FDI compared to usual care for hospitalization with infection as the main cause, hospitalization or death with infection as the main cause, and for hospitalization with infection as the main or contributory cause (*Table* *2*, *Figure* *2*). However, these differences did not reach statistical significance. There were numerically fewer hospitalizations for COVID‐19, other respiratory and skin infections (cellulitis) as the main cause in those assigned to FDI, with similar numbers of urinary and other infections in the two arms (*Table* *2*).

Comparing the results in *Table* *3* (follow‐up to 1 year only) with those in *Table* *2* (follow‐up for the whole trial), the HRs and risk ratios were generally smaller in the first year (that is the impact of FDI appeared to be greater), particularly for the recurrent event analyses that were most likely impacted by the COVID‐19 pandemic. There were significant reductions in the risk of recurrent hospitalization or death with infection as the main cause (rate ratio [RR] 0.52, 95% CI 0.39–0.91, *p* = 0.017), recurrent hospitalization with infection as the main cause (RR 0.59, 95% CI 0.38–0.92, *p* = 0.020), and hospitalization with infection as the main or contributory cause (RR 0.64, 95% CI 0.48–0.83, *p* = 0.0042).

**Table 3:** Hospitalizations with, and deaths due to, infection in the first year only

Outcome	Usual care (*n* = 568)	FDI (*n* = 569)	HR or RR (95%CI)	*p*‐value
**First event**	***n* (% with an event)**	***n* (% with an event)**		
Hospitalization or death with infection as the main cause (first event)	56 (10)	40 (7%)	0.70 (0.46–1.04)	0.079
Hospitalization with infection as the main cause (first event)	53 (9)	37 (7)	0.68 (0.45–1.10)	0.19
Hospitalization with infection as the main or contributory cause (first event)	100 (18)	85 (15)	0.82 (0.62–0.98)	0.038
Deaths with infection as the main cause	6 (1)	8 (1)	1.33 (0.46–3.84)	0.59
**Recurrent events**	***n* (rate per 100 patient‐years)**	***n* (rate per 100 patient‐years)**		
Hospitalizations with infection as the main cause (recurrent events)	71 (13)	42 (8)	0.59 (0.38–0.92)	0.020
Hospitalization or death with infection as the main cause (recurrent events)	74 (14)	44 (8)	0.52 (0.39–0.91)	0.017
Hospitalizations with infection as the main or contributory cause (recurrent events)	158 (30)	102 (19)	0.64 (0.48–0.83)	0.0042

Numbers and percentage with events in the first year of follow‐up, for first events and numbers of events and rates/100 patient‐years for recurrent events with hazard ratios (HR) (for first events) and rate ratios (RR) (for recurrent events) with associated 95% confidence intervals (CI) and *p*‐values comparing ferric derisomaltose (FDI) with usual care.

*Table* *4* contains a post‐hoc subgroup analysis based on baseline TSAT <20% (*n* = 841) versus ≥20% (*n* = 269) for the three time‐to‐first event analyses shown in *Table* *2*. For each outcome there was evidence of a reduction in infection events in the IV FDI arm compared to usual care in the TSAT <20% subgroup. There was no evidence of a difference in the TSAT ≥20% subgroup. However, tests for interaction did not meet statistical significance.

**Table 4:** Post hoc subgroup‐analyses of hospitalization or death from infection (all follow‐up) according to baseline transferrin saturation

Outcome	TSAT <20%		TSAT ≥20%		*p*_interaction_
	HR (95% CI)	*p*‐value	HR (95% CI)	*p*‐value	
Hospitalization or death with infection as the main cause (first event)	0.73 (0.55–0.97)	0.029	1.20 (0.73–1.97)	0.47	0.086
Hospitalization for infection as main cause (first event)	0.70 (0.53–0.94)	0.017	1.13 (0.69–1.87)	0.63	0.10
Hospitalization for infection as main or contributory cause (first event)	0.79 (0.63–0.99)	0.039	1.08 (0.72–1.60)	0.71	0.22

Hazard ratios (HR) and 95% confidence intervals (CI) for the effect of ferric derisomaltose relative to usual care split by levels of transferrin saturation (TSAT) (<20% and ≥ 20%) for time‐to‐first event outcomes.

## Discussion

This analysis shows that patients with heart failure and iron deficiency are prone to a substantial risk of hospitalizations for infection and that IV iron replacement with FDI does not increase the risk of serious infection, and might reduce it, especially when the TSAT is <20%. When analysed across the full timescale of the trial this effect was particularly noticeable in the time‐to‐first event analysis. Since many recurrent events by their nature will have occurred later in the trial, the recurrent event analyses were more likely to be affected by the COVID‐19 pandemic. This argument is supported by the analysis of outcomes in the first year of follow‐up where there was much clearer evidence of a reduction in serious infection‐related events with FDI when analysed as recurrent events, including a 6% absolute reduction in the risk of hospitalization or death with infection as the main cause. A further point is that, if FDI is beneficial in reducing the risk of serious infection, then this will be most evident in patients who are truly iron depleted. There is growing evidence that a TSAT <20% provides strong evidence of iron depletion, while ferritin <100 μg/L in patients with TSAT ≥20% does not. Our subgroup analyses suggesting that reduction in infection risk following IV FDI is restricted to patients with a TSAT <20% support this argument. The reduction in events with FDI appeared to be due to fewer hospitalizations due to respiratory, skin/soft tissue and COVID‐19 aetiology. This finding should be investigated in other studies.

Our current data should be reassuring to clinicians and patients and aid the shared decision‐making process; the benefits of IV FDI do not come at the expense of increased risk of infection. Although not specifically assessed in the current analysis, given that patients with active infection were excluded, most would still agree that IV iron infusions should not be administered during active bacterial infections due to concern that iron is required for pathogen replication. Previous trials of IV iron have generally not pre‐specified infection as an endpoint and as such reporting of infections may have been subject to reporting bias.^9^ In different patient populations, the subtypes of infection will likely differ, and this could also affect the observed overall effect of IV iron on infections. A major strength of our data is that serious infection‐related events were pre‐specified safety endpoints and that all unplanned hospitalizations and all deaths were blindly adjudicated. Our data add to those from the recent PIVOTAL trial of IV iron sucrose in patients with end‐stage kidney disease treated with dialysis.^11^ This trial compared a high dose pro‐active versus lower dose reactive IV iron dosing regimen and found no evidence of a dose response effect in terms of infection risk, but in line with international dialysis practice ferritin levels in the pro‐active arm were substantially greater than in IRONMAN.^11^ Intravenous iron preparations differ with respect to the amount of free iron; FDI has tight binding of iron within the carbohydrate complex, with very low levels of labile iron.^17^, ^18^ It would be interesting to compare the effect of FDI on infection with that other IV iron preparations which do not have this property.

The COVID‐19 pandemic profoundly affected the conduct of the IRONMAN trial; at times, in‐person review of research patients was not permitted preventing assessment of iron status and, if indicated, re‐dosing with FDI, which could have led to failure to maintain iron repletion. In addition, as the trial progressed, more patients assigned to usual care received IV iron outside the protocol. Such protocol deviations could obscure both beneficial and harmful effects of correcting iron deficiency. First event analyses or censoring follow‐up at 1 year reduce the confounding effects of the pandemic, not only providing more robust evidence of safety of IV FDI but also suggesting greater benefit. Recent trials of IV iron in heart failure have used the criteria for iron deficiency recommended in heart failure guidelines but these are not based on robust scientific evidence. This allowed inclusion of patients with a ferritin of <100 μg/L irrespective of other iron markers. A TSAT <20% may be a better marker of response to IV iron in patients with heart failure^14^, ^19^, ^20^ and the reduction in infection with FDI seemed clearest for this group of patients.

It is not possible to infer from our data that IV iron reduces the risk of infection, more that it may reduce the risk of serious infection that causes or contributes to hospitalization or death in those with heart failure and iron deficiency. This could be because correction of iron deficiency makes patients more resilient to an insult, in this case exposure to infection. However, it is plausible that there are more specific mechanisms involved given iron has an important role in the immune response, and iron deficiency is associated with reduced T cell and macrophage function and reduced bactericidal capacity of polymorphs.^6^, ^21^, ^22^, ^23^ The bioavailability of iron to cells is reflected by how much iron is bound to transferrin in the circulation and this is represented by TSAT. The finding that patients with a TSAT <20%, a marker of reduced iron bioavailability, exhibited a significant reduction in the risk of serious infection with IV FDI adds further support. Of note, infection was also a common contributary cause for hospital admission. It is uncertain how precisely these infections impacted admissions and whether a reduction in contributary infections can be interpreted as causally due to treatment. Nevertheless, the high rate of contributary infections emphasizes the complexity of typical heart failure hospitalizations.

Respiratory infections were common but numerically fewer in patients randomized to FDI. Differentiating respiratory infection from worsening heart failure may be clinically challenging, and the two conditions often co‐exist.^12^ Several other effective treatments for heart failure also reduce the risk of respiratory infections.^24^, ^25^ It is plausible that some participants may have been adjudicated as presenting with heart failure when they had infection, and vice versa. However, we found evidence that FDI decreased the risk of hospitalization due to heart failure as well as hospitalization with infection.^3^ The COVID‐19 pandemic placed patients with heart failure at a higher risk of complications and mortality than patients without heart failure. In this trial, FDI was associated with numerically fewer COVID‐19 related hospitalizations and deaths. Total numbers of severe COVID‐19 events were relatively low, but this merits further evaluation for patients with heart failure and/or other comorbidities associated with iron deficiency. Replenishing iron helps the immune response,^26^ whilst low serum iron seems to impair the response to immunization,^24^ for example blunting the T cell, B cell and neutralizing antibodies to influenza vaccine.^6^ We do not have data on COVID‐19 immunization for participants in the IRONMAN trial, but there is no reason to believe that the high uptake of vaccination seen in the UK would differ between the arms of the trial.

There are limitations within the current analysis. The population was mainly Caucasian and as such extrapolation of the results to other ethnic populations should be done with caution. With a median age of around 74 years and with frequent comorbidities the population reflects patients encountered in routine clinical practice. The infection‐related events recorded were those that resulted in hospitalization or death and hence we are unable to comment on the number or type of infections that were managed outside of hospital. It was not possible to be certain of the precise nature of infection in all patient events. Whilst over two thirds (69%) were respiratory (separately reporting COVID‐19), skin and soft tissue, and urinary, the site of infection could not always be specified. This clinical scenario is frequently encountered in clinical practice, with patients receiving broad spectrum antibiotics in the face of pyrexia and/or elevated inflammatory markers. We are not able to comment on specific inflammatory markers since they were not routinely collected from these serious adverse events.

## Conclusion

This analysis from the IRONMAN trial found that patients with heart failure and reduced or mildly reduced LVEF and iron deficiency are prone to a substantial risk of serious infection. IV FDI is not associated with an increased risk of hospitalization or death from infection, and may in fact be protective in this population, especially when TSAT is <20%.

## Supporting information


**Appendix S1.** Supporting Information.
